# First insights into the pleiotropic role of *vrf* (*yedF*), a newly characterized gene of *Salmonella* Typhimurium

**DOI:** 10.1038/s41598-017-15369-7

**Published:** 2017-11-10

**Authors:** Clara Ballesté-Delpierre, Dietmar Fernandez-Orth, Mario Ferrer-Navarro, Ramón Díaz-Peña, Antonia Odena-Caballol, Eliandre Oliveira, Anna Fàbrega, Jordi Vila

**Affiliations:** 1 ISGlobal, Barcelona Centre for International Health Research (CRESIB), Hospital Clínic - Universitat de Barcelona, Barcelona, 08036 Spain; 2 Plataforma de Proteómica, Parc Científic de Barcelona, 08028 Barcelona, Spain; 3 Present Address: ISGlobal, Centre for Research in Environmental Epidemiology (CREAL), 08003 Barcelona, Spain; 4 Present Address: Department of Clinical Microbiology, Hospital Vall d’Hebron, Vall d’Hebron Institut Recerca (VHIR), Universitat Autònoma de Barcelona, 08035 Barcelona, Spain

## Abstract

*Salmonella* possesses virulence determinants that allow replication under extreme conditions and invasion of host cells, causing disease. Here, we examined four putative genes predicted to encode membrane proteins (*ydiY, ybdJ*, *STM1441* and *ynaJ*) and a putative transcriptional factor (*yedF*). These genes were identified in a previous study of a *S*. Typhimurium clinical isolate and its multidrug-resistant counterpart. For STM1441 and *yedF* a reduced ability to interact with HeLa cells was observed in the knock-out mutants, but an increase in this ability was absent when these genes were overexpressed, except for *yedF* which phenotype was rescued when *yedF* was restored. In the absence of *yedF*, decreased expression was seen for: i) virulence-related genes involved in motility, chemotaxis, attachment and survival inside the host cell; ii) global regulators of the invasion process (*hilA*, *hilC* and *hilD*); and iii) factors involved in LPS biosynthesis. In contrast, an increased expression was observed for anaerobic metabolism genes. We propose *yedF* is involved in the regulation of *Salmonella* pathogenesis and contributes to the activation of the virulence machinery. Moreover, we propose that, when oxygen is available, *yedF* contributes sustained repression of the anaerobic pathway. Therefore, we recommend this gene be named *vrf*, for virulence-related factor.

## Introduction

*Salmonella* Typhimurium is an enteric food-borne pathogen responsible for causing salmonellosis associated with acute diarrhea. The bacterial invasion initially requires flagellar motility to reach the intestinal lumen and to cross the mucus layer of the intestinal epithelia and subsequent adhesion to the host cell, mainly mediated by fimbriae^1^.

The key genes involved in the pathogenic process are encoded within highly conserved regions of the bacterial genome called *Salmonella* Pathogenicity Islands (SPIs). The key regulator HilA, which is positively regulated by HilC and HilD, is located in SPI-1; however, other virulence-related regulators, such as RtsA, are encoded outside this island^2^. During the invasion, proteins encoded by the *prgHIJK*, *spaMNOPQRS*, and *invABCEFGH* SPI-1 operons constitute a Type-3 Secretion System. Effector proteins, such as SipA and SipC (encoded in SPI-1) and SigD/SopB (encoded in SPI-5), translocate to the host cytosol through this system causing intracellular changes and inducing the immune response^2–4^. The survival and replication of intracellular *Salmonella* inside *Salmonella*-containing vacuoles (SCVs) is mainly mediated by the genes located in SPI-2^3^. The immune response is activated through the recognition of pathogen-associated molecular patterns (PAMPs) by specific receptors expressed on the surface and/or inside different cell types^1,5,6^. The most important PAMPs are flagellin (monomers comprising the flagella filaments), particularly the FliC subunit, which is highly expressed on the bacterial surface, and lipopolysaccharide (LPS)^7^.

Oxygen availability is limited in the inflamed gut whereas other compounds, such as hydrogen sulfide (H_2_S)^8^ and H_2_^9^ are abundantly produced by the colonic bacteria. In addition, nutrients are also limited inside the SCV, while reactive oxygen as well as nitrogen species may be found^10^. In anaerobic conditions, *Salmonella* is able to survive by using alternative energy sources, such as nitrate or fumarate, through the use of specific enzymes: nitrate and nitrite reductases^11^, fumarate reductase, DMSO reductase^12,13^ and respiratory hydrogenases^9,14^.

In a previous study, we compared transcriptomes of a clinical isolate of *S*. Typhimurium (strain 50-wt) and its derivative-multidrug-resistant mutant (strain 50–64) using microarrays^15^. In addition to showing antimicrobial resistance, 50–64 strain was also less invasive. In the present study, we focused on the genes of unknown function that showed impaired expression. For genes *ybdJ*, *STM1441* and *ynaJ* a higher expression was seen in 50–64 compared to the wild-type strain (33.2-, 5.7- and 3.05-fold, respectively) whereas transcription was reduced in 50–64 for genes *ydiY* and *yedF* (−5.4- and −4-fold, respectively). We further selected those genes with a putative role in the acquired resistance phenotype or in the repressed virulence observed. Here, 4 putative membrane proteins and the *yedF* of unknown function were investigated to evaluate their involvement in these two phenotypes.

## Results

Four genes potentially related to efflux (annotated as putative inner -*ybdJ, STM1441* and *ynaJ-* and outer -*ydiY-* membrane proteins) were selected from a previous work for their differences at a transcriptional level^15^. In this work, an antibiotic resistant *S*. Typhimurium strain (50–64) showing *in vitro* impairment of the ability to be internalized in HeLa cells was compared to its susceptible counterpart (50-wt). Mutants of the reference strain SL1344 either carrying the disrupted genes or overexpressing them were obtained. Our experiments revealed that none of the genes were linked to antimicrobial susceptibility (data not shown) and statistically significant differences in the ability to invade HeLa cells were seen for both ∆*STM1441* and the strain overexpressing this gene (STM1441_pBAD33). However, these results are contrary to what was expected as the poorly virulent mutant (50–64) showed higher transcriptional levels of *STM1441* than 50-wt (Supplementary Figure 1). These inconclusive and contradictory results led to discontinuation of the study of these genes.

Another novel gene selected was the *S*. Typhimurium homolog of the *E. coli yedF* gene. Predicted to encode a TusA-like protein of unknown function, it has a 31% homology to the *E. coli* gene based on sequence alignment^16^. TusA is a sulfur transfer protein involved in tRNA modification and molybdenum cofactor biosynthesis in *E. coli* ^16,17^. From a structural point of view, the N-terminal region of *yedF* contains a CPxP conserved motif and the C-terminal domain has a similar folding structure to the translation initiation factor IF3C of *E. coli*. Together, these observations support the idea of a possible involvement of *yedF* in mRNA binding^18,19^. As seen for the other genes, neither mutants lacking *yedF* nor those overexpressing it showed any change in the antimicrobial susceptibility profile.

### *In vitro* ability to interact with HeLa cells is compromised in the mutant lacking *yedF*

The deletion mutant lacking *yedF* (Δ*yedF*) showed a statistically significant 3.25-fold reduction in its ability to adhere/invade HeLa cells (p = 0.049) relative to the reference strain SL1344 (8.21% *vs* 26.64%, respectively). Furthermore, overexpression of *yedF* led to an increased ability to interact with the eukaryotic cells above the levels of the reference strain SL1344_pBAD33 (45.17% *vs* 33.40%, respectively), despite the difference not being statistically significant. These results were in line with the phenotype seen in 50–64 strain, suggesting the involvement of *yedF* in the virulence-associated phenotype.

Complementation of *yedF* in the Δ*yedF* background was not possible using the pBAD33 vector as the antibiotic used for the selection in both systems was chloramphenicol. For this reason, the gentamicin protection assay was repeated with a new collection of mutants using the p9817 vector conferring ampicillin resistance (Supplementary Table 1). Previously, the lack of expression of the gene *yedF* in the strains Δ*yedF* and its overexpression in the complemented strain Δ*yedF*p9817*yedF* (fold-change = 1642,44; pvalue < 0.01) compared with the wild-type strain SL1344 were confirmed by RT-PCR.

**Figure 1:**
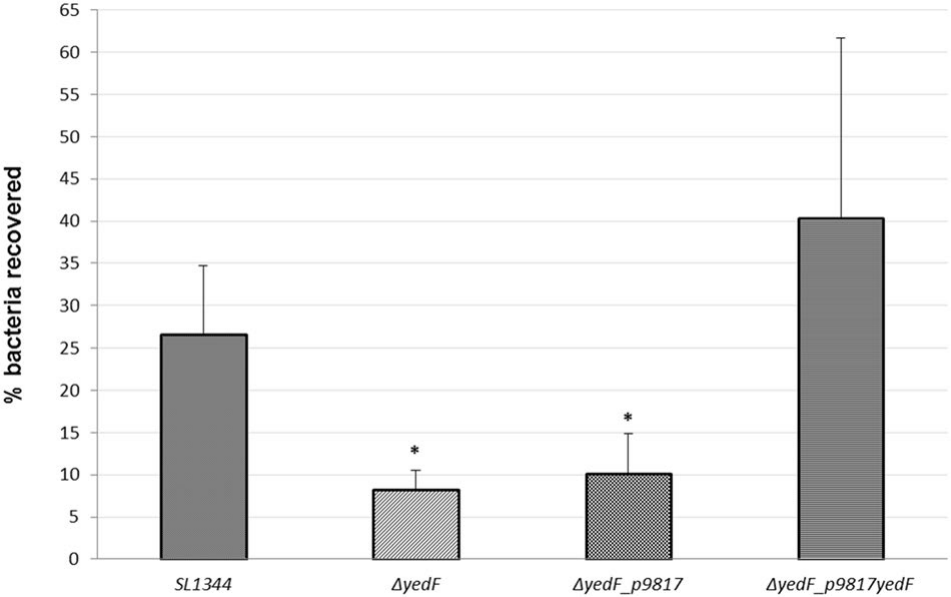
Percentage of bacteria able to interact with HeLa cells for the reference strain (SL1344), mutant lacking *yedF* (∆*yedF)*, mutant lacking *yedF* but carrying the empty plasmid (∆*yedF_p9817)* and the complemented strain (∆*yedF* _p9817*yedF*). The strain ∆*yedF* was obtained using the Datsenko & Wanner method by replacing the coding region of the gene *yedF* by a chloramphenicol-resistant cassette, and further transforming the empty vector p9817. The mutant lacking *yedF* was complemented in *trans* with p9817, a high copy number plasmid, carrying the gene of interest. The gentamicin protection assay performed consisted on infecting HeLa cells with the different strains (exponential cultures grown at 37 °C without shaking) at a multiplicity of infection of 100. The percentage of bacteria able to interact with HeLa cells was calculated as the ratio between the bacteria load used to infect eukaryotic cells and the amount of bacteria recovered at the end of the assay, being these results the mean of 3 independent experiments with standard deviation. One asterisk indicates a pvalue < 0.05.

Then, the gentamicin protection assay was conducted and the results obtained were consistent with the previous findings; namely, the absence of *yedF* (for both Δ*yedF* and the mutant carrying the empty plasmid, Δ*yedF_p9817*) led to a statistically significant reduction (−3.24-fold and −2.62-fold, respectively) in the ability to interact with HeLa cells relative to the reference strain (8.21% and 10.16% *vs* 26.64%). When the gene was reintroduced, the capability of this strain to be internalized by epithelial cells was even greater than the reference strain achieving 40.32% (Fig. 1). This effect was likely due to the promoter used on the exogenous vector or to the copy number of the plasmid.

### Transcriptomic analysis reveals that virulence-related genes are affected by *yedF*

A transcriptomic comparison between the SL1344 strain and ∆*yedF*, using the RNA-Seq, revealed that in the deletion strain 630 genes were repressed and 321 genes were overexpressed (p < 0.05). The complete list of genes with the corresponding fold-change values and a hierarchical cluster with the significant genes are reported as S1 Supplementary Data and Supplementary Figure 2, respectively.

**Figure 2:**
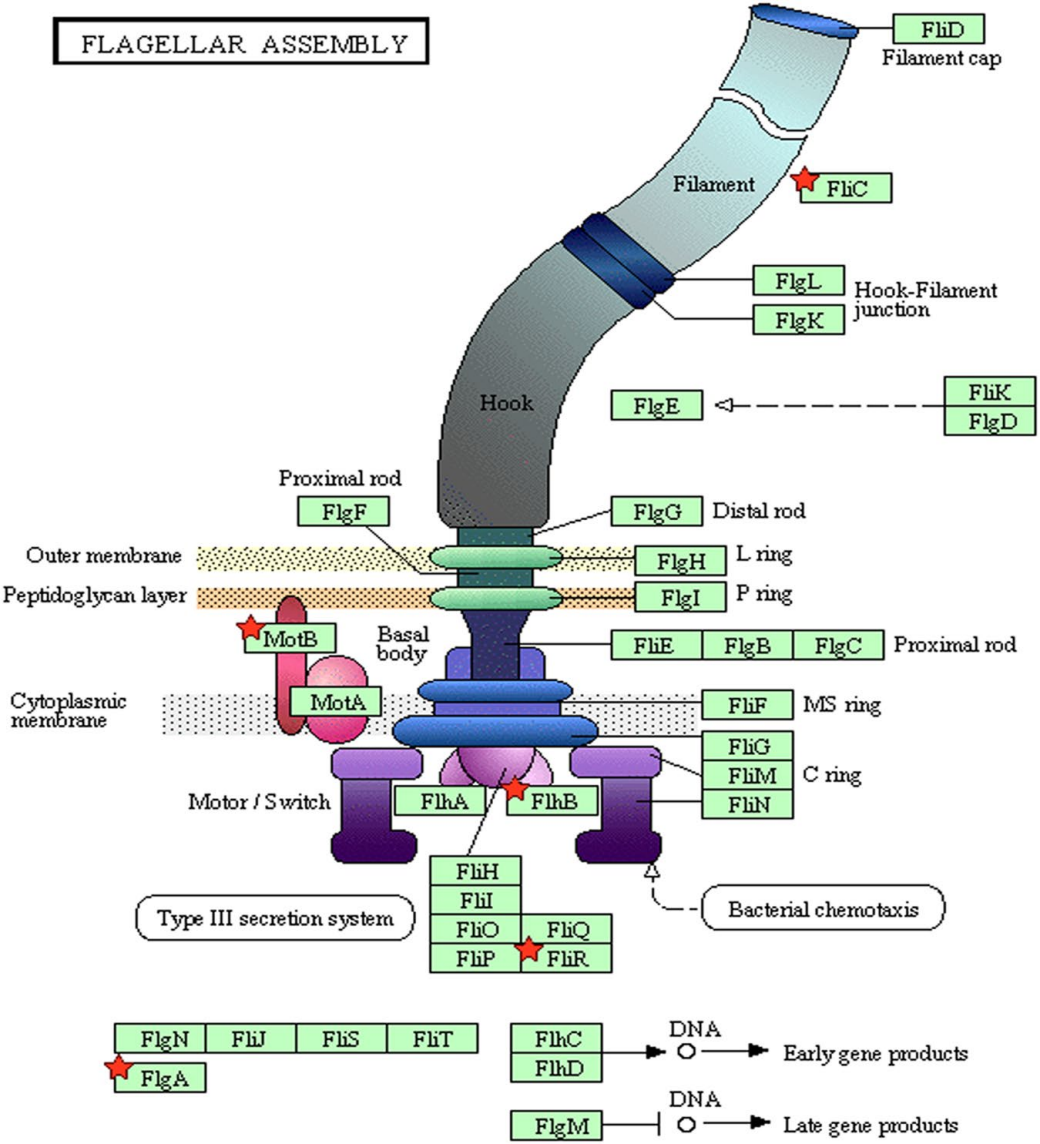
Representation of the structure of the flagellum. This image represents the different components that constitute the flagellum (modified from the KEGG PATHWAY Database^49^). Red symbols indicate differentially expressed genes between the SL1344 strain and its mutant lacking *yedF* detected in the RNA-Seq assay.

**Figure 3:**
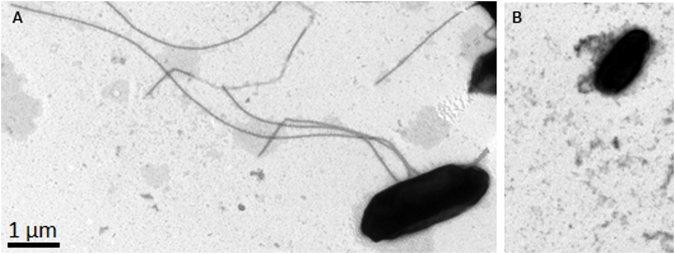
Electron microscopy of the wild-type strain SL1344 and the mutant lacking *yedF* (∆*yedF)*. Negative staining preparation of both samples was performed and visualization by Transmission Electron Microscopy (TEM) through a TEM JEOL 1010 equipped with a camera CCD Orius (GATAN) at an operating voltage of 80 kV. Images obtained revealed the absence of flagella in the mutant strain (**B**) whereas SL1344 showed intact flagella (**A**).

In accordance with the results obtained from the gentamicin protection assay, a significant number of the repressed genes seen in ∆*yedF* were related to virulence. With respect to motility, the flagellum-related genes encoding for the late flagellin proteins FliC, the type III secretion apparatus FliR, and FlgA, involved in the flagellar assembly^20,21^ were repressed in ∆*yedF* relative to SL1344 (fold change of −2.17, −2.77 and −2.41, respectively) (Fig. 2 **)**. Indeed, electron microscopy experiments revealed that ∆*yedF* was aflagellated whereas SL1344 did show flagella on its surface (Fig. 3). However, this downregulation observed of *fliC*, together with the transcriptional repressor *fljA* and the phase 2 flagellin gene *fljB* (−11.48- and −5.97-fold, respectively) is contradictory. When flagellar phase variation is induced, FljA is expressed and inhibits the translation of the FliC mRNA by binding to its operator region. This results in the transcription of FljB and the absence of FliC^22–24^.

In addition to motility, several chemotaxis genes required during the initial invasion^2^ were also down-regulated in ∆*yedF*. This was also the case for the type-1 fimbrial gene *fimA* and the regulatory gene *fimZ* (−5.1- and −3.07-fold, respectively).

Moreover, a broad repression of the genes encoded in the SPIs involved in the host invasion process (SPI-1, SPI2, SPI-4 and SPI-5) was also seen. Accordingly, significant repression was observed for the key regulators *hilA* and *hilC* (−3.10 and −3.57, respectively) in the *yedF*-deficient strain although repression was non-statistically significant for *hilD* (−1.89) (Supplementary Data).

**Figure 4:**
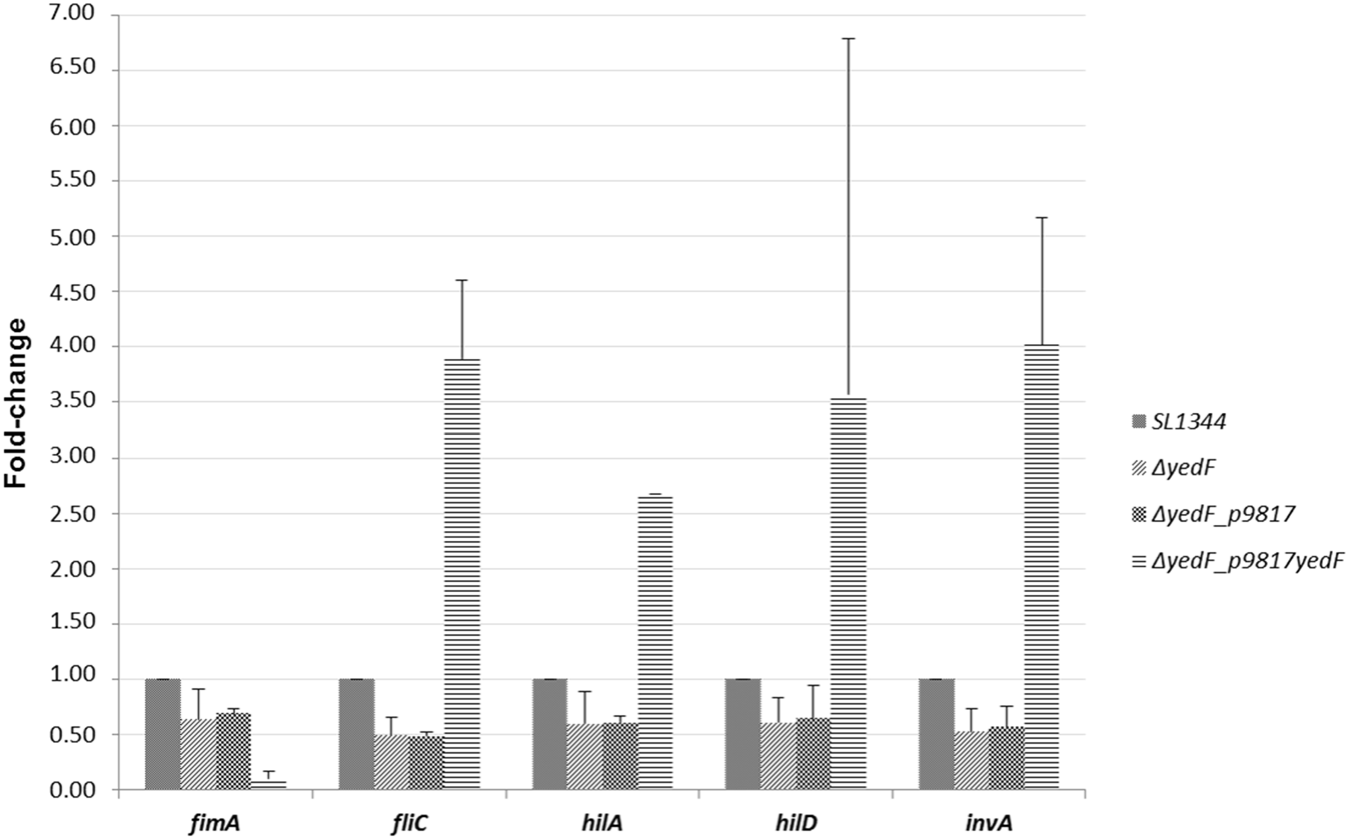
Expression levels of the genes involved in virulence in the wild-type (SL1344), strain lacking *yedF* (∆*yedF)*, mutant lacking *yedF* but carrying the empty plasmid (∆*yedF_p9817)* and the complemented strain (∆*yedF*_p9817*yedF*). RT-PCR of selected genes involved in virulence (*fimA*, *fliC*, *hilA*, *hilD* and *invA*) was performed for these four strains revealing that in all cases, the strains lacking *yedF* showed a reduction in the transcriptional levels compared with the reference strain (SL1344). When the wild-type gene was restored (as was the case in the complemented strain ∆*yedF*_p9817*yedF)* gene expression of all but one determinant (*fimA*) was increased above the levels of the SL1344. Transcription levels are represented as the fold-change calculated as the ratio between the value of relative quantitation of the mutants compared to the reference strain, SL1344, which value is 1 (mean of 3 independent experiments with standard deviations).

To further investigate the involvement of *yedF* in virulence gene transcription, RT-PCR of *fliC*, *fimA*, *hilA*, *hilD* and *invA* was carried out in the ∆*yedF* strain complemented with p9817 (*yedF*) and compared to both the reference strain and the ∆*yedF* mutant. As expected, the expression levels of these virulence genes were restored, reaching even higher values than those observed for the reference strain SL1344_p9817 (Fig. 4). Surprisingly, 10-fold repression was observed for the fimbria-encoding gene *fimA*, which is significantly higher than repression seen in the knock-out strain (−1.45-fold). A possible explanation could be that *yedF* is a positive regulator of *fimA* transcription and excessive amounts of the regulator provoke a paradoxal opposite effect.

**Figure 5:**
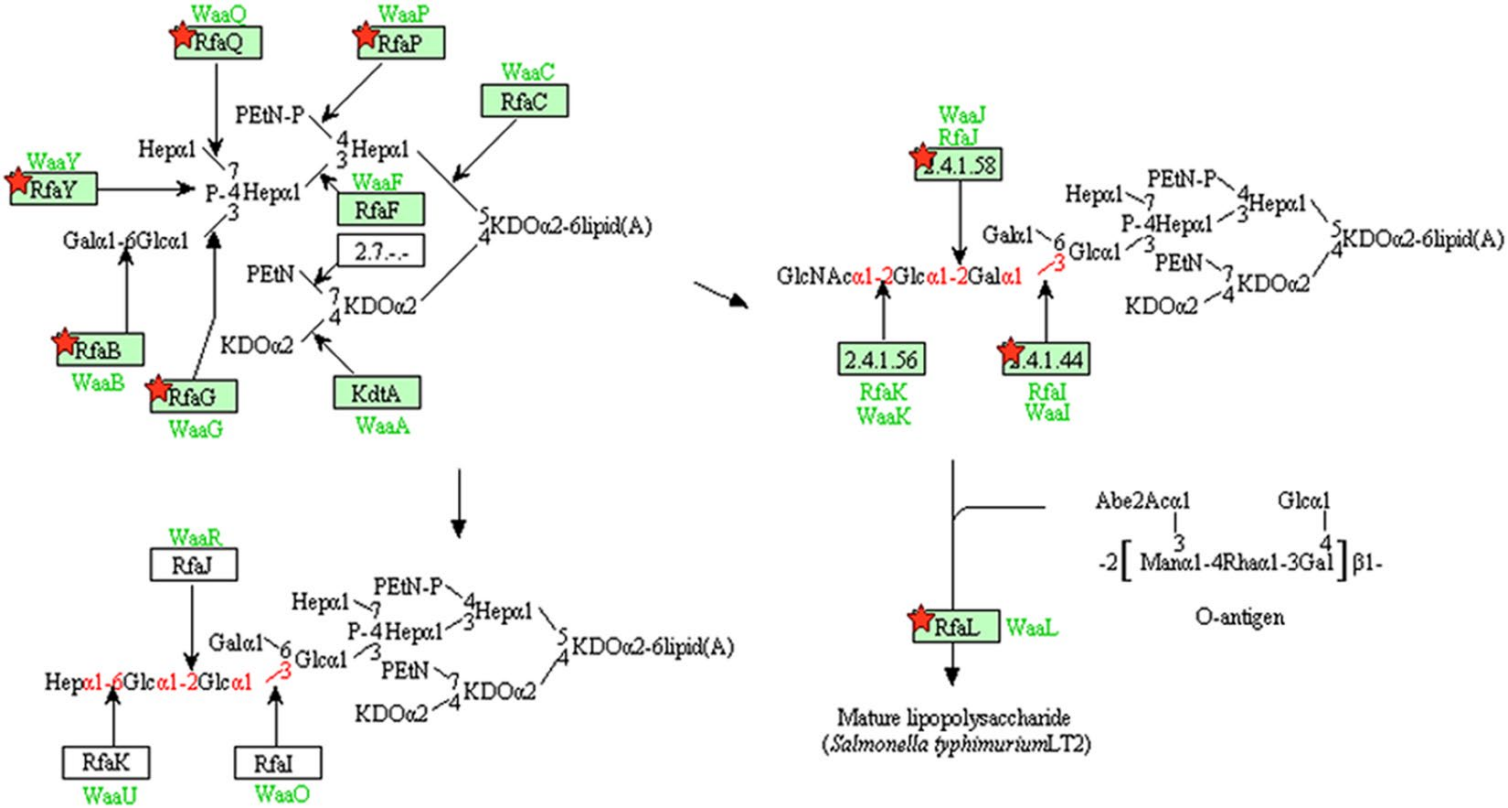
Representation of the LPS biosynthetic pathway. This image represents the different determinants involved in the biosynthesis of LPS (modified from the KEGG PATHWAY Database^49^). Components marked with red symbols indicate differentially expressed genes between the SL1344 strain and its mutant lacking *yedF* detected in the RNA-Seq assay.

Interestingly, genes involved in LPS biosynthesis, another relevant virulence determinant, were also repressed in *∆yedF* (see Supplementary Data). LPS can be recognized by specific receptors of the host cells promoting innate inflammation pathways^5,6^. Transcription of the genes involved in O-antigen polysaccharide assembly (*rfb* genes) as well as in core-oligosaccharide composition (*rfa* genes)^25,26^ were repressed in *∆yedF* (Fig. 5), with values ranging from a −2.03- to −6.79-fold change relative to SL1344. To validate the transcriptomic data, LPS content was measured. As expected, a decrease in the concentration of endotoxins was observed in the knock-out strain (*∆yedF)* compared to SL1344 (5.1 × 10^4^ EU/mL *vs* 7.52 × 10^4^ EU/mL). Similar results were seen when the endotoxin levels were checked in *∆yedF* _p9817, the mutant carrying the empty plasmid (4.99 × 10^4^ EU/mL). These levels increased to 6.46 × 10^5^ EU/mL when *yedF* expression was restored by complementation, being all differences observed between the strains statistically significant (p = 0.05). Standard deviations for SL1344, *∆yedF, ∆yedF*_p9817 and the complemented strain (*∆yedF* _p9817*yedF)* were 137.5, 115.4, 57.7 and 251.7, respectively.

### *yedF* is involved in transcriptional regulation of the anaerobic metabolism pathway

Transcriptomic analysis revealed that all genes overexpressed in ∆*yedF* relative to SL1344 encoded for proteins involved in anaerobic respiration (Table 1). In the absence of oxygen, *Salmonella* is able to survive by performing fermentations and/or by using alternative electron acceptors such as nitrate or fumarate^13^. Specifically, the genes overexpressed in the absence of *yedF* included: the cytoplasmic nitrite reductase NirB encoding genes (almost 40-fold increased expression), the nitrate reductase encoded by the *narGHIJ* operon (8- to 30-fold), the anaerobic DMSO reductase (almost 15-fold), and the *nrdDG* operon encoding for the class III reductase NrdD (12.5-fold). While the membrane-bound nitrate reductase NarGHI has shown to be responsible for NO production in NO^−^_3_ conditions in *Salmonella* ^27^, the involvement of the cytoplasmic nitrite reductase NirB in NO production has only been previously demonstrated in *E. coli* ^28^ but not in *S*. Typhimurium^27^. Additionally, the genes encoding for the formate dehydrogenase H (FdhF), a component of the formate hydrogenlyase (FHL) complex, and the genes encoding for the maturation proteins HypABCDE that together catalyze the production of hydrogen from formate ^28^ were also activated in *∆yedF* (2.67- to 10.38-fold).The transcriptional activator of the *fdhF* gene, named *fhlA*, was also up-regulated by 5.33-fold as was the *glpABC* operon, encoding for the anaerobic glycerol-3-phosphate dehydrogenase subunits (2.56- to 5.28-fold). In *E. coli* this enzyme has been reported to be essential for anaerobic growth on glycerol-3-phosphate^29,30^.

**Table 1:** Transcriptional values of representative genes overexpressed in the *∆yedF* mutant compared to the wild-type SL1344. Fold change indicate the ratio between the expression levels in SL1344 and *∆yedF*. For RNA-Seq, only statistically significant results (p < 0.05) are represented.

Enzymes and related genes	Locus tag	Gene	Description	Fold change (*∆yedF*/ SL1344*)*
Nitrate/nitrite reductase				
	SL1344_1689-SL1344_1695	*narIJHGKXL*	respiratory nitrate reductase	2.07–30.19
	SL1344_2225-SL1344_2231	*napCBHGADF*	periplasmic nitrate reductase	3.46–12.09
	SL1344_2443	*narQ*	nitrate/nitrite sensor protein NarQ	4.08
	SL1344_3441-SL1344_3443	*nirBDC*	nitrite reductase	8.98–38.90
	SL1344_4213-SL1344_4217	*nrfABCDE*	Nitrite reductase complex	1.34–5.16
Fumarate reductase				
	SL1344_4277-SL1344_4280	*frdDCBA*	fumarate reductase	3.40–4.76
Anaerobic dimethyl sulfoxide reductase				
	SL1344_0902	*dmsA*	anaerobic dimethyl sulfoxide reductase subunit A	16.37
	SL1344_1428	*dmsA2*	putative dimethyl sulfoxide reductase subunit	14.79
	SL1344_4242	—	anaerobic dimethyl sulfoxide reductase chain	7.42
Anaerobic ribonucleoside reductase				
	SL1344_4381-SL1344_4382	*nrdGD*	anaerobic ribonucleoside-triphosphate reductase activating protein	3.21–12.49
Anaerobic glycerol-3-phosphate dehydrogenase				
	SL1344_2253-SL1344_2255	*glpABC*	anaerobic glycerol-3-phosphate dehydrogenase	2.56–5.28
Hydrogenase				
	SL1344_2833	*hycA*	formate hydrogenlyase regulatory protein	4.82
	SL1344_2834-SL1344_2838	*hypABCDE*	Hydrogenase maturation proteins	2.67–10.38
	SL1344_2839	*fhlA*	transcriptional activator of the formate hydrogenlyase system	5.33
	SL1344_3118-SL1344_3124	*hybFEDCBA*	hydrogenase-2	2.04–6.49
	SL1344_4221	*fdhF*	putative formate dehydrogenase H	4.16

The transcriptional up-regulation of all the genes involved in anaerobic metabolism seen in the present investigation is in accordance with a previous study conducted in *E. coli* ^16^. In that work, deletion of the sulfur transferase *tusA* triggered a similar situation to that observed here for hydrogenases and the already mentioned *nar* operon as well as *nap* operon in aerobic conditions. In the absence of *tusA*, the expression of the corresponding enzymes was increased suggesting that *yedF*, which was previously mentioned to be partially homologous to *tusA*, has a similar role in the regulation of these proteins in *S*. Typhimurium.

**Figure 6:**
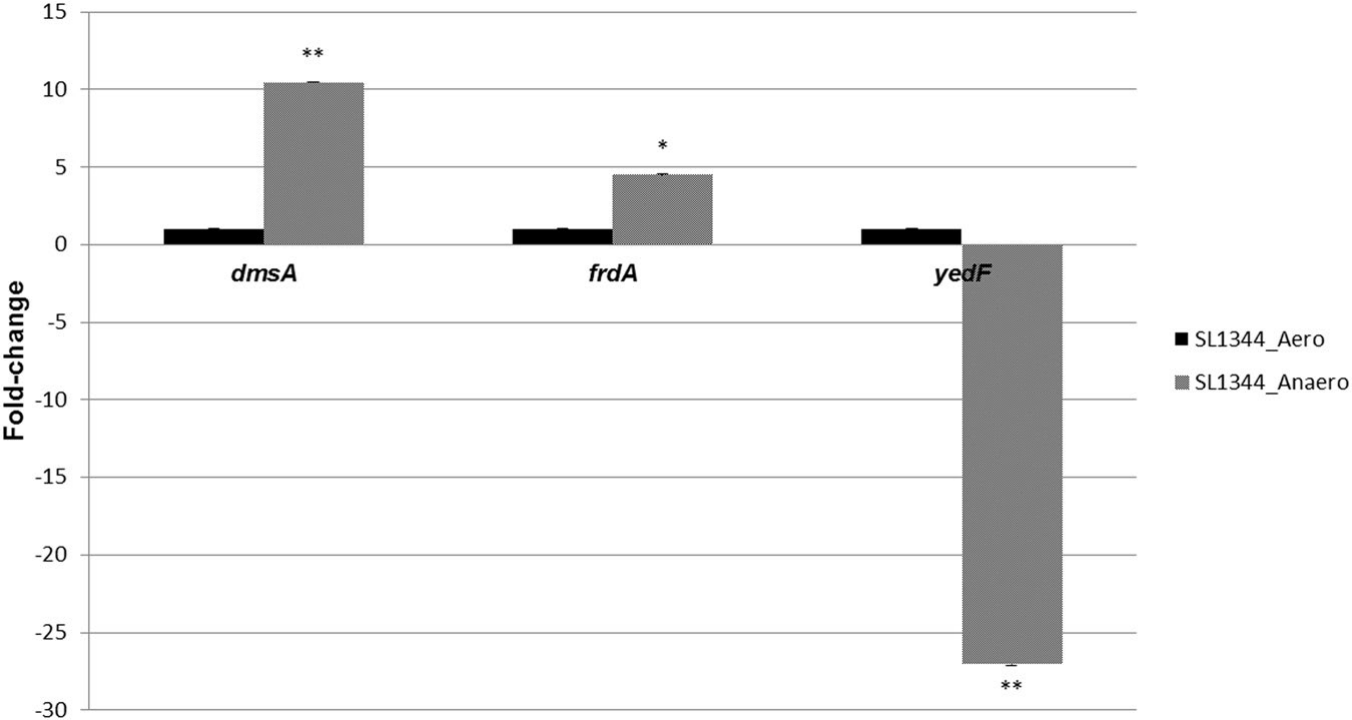
Expression levels of *dmsA* and *frdA*, representative for the anaerobic metabolism, and *yedF* in the reference strain SL1344. RT-PCR of the genes *dmsA*, *frdA* and *yedF* was performed from the strain SL1344 obtained from exponential cultures in both aerobic and anaerobic conditions in three independent experiments. The results obtained indicate that an overexpression of the genes *dmsA*, encoding the subunit A of the anaerobic dimethyl reductase, and *frdA*, part of the fumarate reductase operon, occurred in the absence of oxygen, being these differences statistically significant. Moreover, the expression of *yedF* was seen to be repressed when oxygen was not available. These results indicate that under anaerobic conditions the expression of *yedF* is repressed whereas an activation of the expression of the anaerobic metabolism of *Salmonella* occurs. Transcription levels are represented as the fold-change calculated as the ratio between the value of relative quantitation of SL1344 in anaerobic conditions compared to the same strain in the presence of oxygen, which value is 1 (mean of 3 independent experiments with standard deviations). One asterisk indicates a pvalue = 0.05–0.01, and two asterisks a pvalue < 0.01.

We further checked by RT-PCR the expression levels of the genes *dmsA* and *frdA* as representatives of the anaerobic metabolism, as well as the expression levels of *yedF* in the presence and absence of oxygen in the reference strain SL1344 (Fig. 6). Both anaerobic-related genes (*dmsA*, encoding the subunit A of the anaerobic dimethyl reductase, and *frdA*, part of the fumarate reductase operon), were statistically significant overexpressed in the absence of oxygen (10.42- and 4.50-fold change, respectively) compared to aerobic conditions. Moreover, a statistically significant repression of the expression of *yedF* was observed (−27.04-fold change) when oxygen was not present. These results are in accordance with the transcriptomic findings described in this section.

### The production of proteins related to virulence and anaerobic metabolism is decreased in the absence of *yedF*

The proteomic approach revealed that 13 out of 16 proteins repressed in Δ*yedF* were related to pathogenesis: 6 were involved in chemotaxis, 3 were flagellar-related proteins (FlgE, FliG and FliC), 2 fimbrial proteins (FimA and FimC), and 2 were the invasion-related determinants SipC and SigD.

The remaining 3 proteins identified as being expressed at lower levels in Δ*yedF* relative to SL1344 belonged to the anaerobic metabolism. There were: i) the glycyl radical cofactor, produced only in the absence of oxygen by the class III ribonucleotide reductases (RNRs)^31^, ii) the ornithine decarboxylase (or SpeF) which buffers the extracellular environment under acidic conditions and has recently been demonstrated to act only in anoxic environments^32^ and iii) the formate dehydrogenase α-subunit, a member of the formate hydrogenlyase (FHL) complex, activated under anaerobic conditions^33^ (Table 2).

**Table 2:** Proteins with decreased production levels in the *∆yedF* mutant compared to the wild-type strain SL1344.

Locus tag	Protein	Description	Fold change (∆*yedF/* SL1344*)*
Flagella			
SL1344_1114	FlgE	flagellar hook protein FlgE	−2.40
SL1344_1888	FliC	flagellin	−5.44
SL1344_1899	FliG	flagellar motor switch protein FliG	−2.03
Chemotaxis			
SL1344_1856	CheA	chemotaxis protein	−2.52
SL1344_1855	CheW	purine binding chemotaxis protein	−2.24
SL1344_3189	—	chemotaxis protein	−2.03
SL1344_1850	CheZ	chemotaxis protein	−2.01
SL1344_2283	CheV	chemotaxis protein	−4.78
SL1344_4464	—	methyl-accepting chemotaxis protein	−3.54
Fimbriae			
SL1344_0536	FimA	type-1 fimbrial protein, a chain	−4.70
SL1344_0538	FimC	fimbrial chaperone protein	−4.26
Invasion processes			
SL1344_1030	SigD	cell invasion protein	−2.12
SL1344_2863	SipC	pathogenicity island 1 effector protein	−5.69
Anaerobic processes			
SL1344_2610	—	glycyl radical cofactor	−5.93
SL1344_0683	SpeF	ornithine decarboxylase	−2.63
SL1344_1500	FdNG	formate dehydrogenase subunit alpha	−2.50

A partial correlation was found upon comparing the proteomic and transcriptomic analyses. All but 2 proteins identified as being expressed less in Δ*yedF* were also seen to be transcriptionally repressed; however, the differences were not statistically significant for half of them. The 2 proteins showing discordant results between the two techniques were the α-subunit of the formate dehydrogenase and the glycyl radical cofactor. Disparity between transcriptomic and proteomic data is often observed as there is no direct correlation between the levels of mRNA and protein expression.

## Discussion

The studies of the four putative outer and inner membrane proteins have not led to conclusive results regarding their role in the phenotypes of antimicrobial resistance or virulence. This could be due to the fact that they play a minor role in efflux and an alteration of these proteins is not sufficient to see a relevant phenotype. Another explanation could be that they may be unrelated to virulence and/or antimicrobial resistance and are rather a collateral effect derived from the adaptation of the bacteria to antibiotic pressure.

More importantly, the present work revealed new insights into the role of the *yedF* gene and shed light on the different *S*. Typhimurium pathways in which this gene is potentially involved. Homology with the *E. coli* TusA suggests that YedF may be a TusA-like protein and that both genes could share at least some of the same functions^19^. In *E. coli*, TusA functions as a sulfur transferase delivering sulfur from the IscS protein to diverse molecules involved in a variety of pathways^16,34,35^. In addition, a previous study showed that an *E. coli* mutant lacking TusA presented severe growth defects^36,37^, suggesting its involvement in the general physiology of the bacteria. However, this function could not be attributed to *yedF* in *S*. Typhimurium as no growth alterations were detected in our Δ*yedF* mutant.

In our case, deletion of *yedF* triggered an increase in gene expression of enzymes involved in anaerobic respiration, similar to what has been demonstrated for *tusA* deletion mutant of *E. coli* ^16^. In this previous study on *E. coli*, *yedF* was also investigated and nitrate reductase activity was tested in both aerobic and anaerobic conditions using a strain lacking *tusA*, *yedF* and a triple mutant lacking these genes in addition to another TusA-like gene (*yeeD*). Neither *yedF* nor *yeeD* were able to replace, even partially, the functions of TusA^16^. However, according to our results, *yedF* seems to play a relevant role in the regulation of proteins involved in anaerobic metabolism in *S*. Typhimurium; although, another TusA-like gene with a protein sequence identity of 90% is also present in this bacterium (GI:486186069). Further studies using single and double mutants of both genes are needed to evaluate the contribution of both the *tusA* homolog and *yedF* in *Salmonella*.

Interestingly, we observed that all of the genes showing transcriptional activation in Δ*yedF* mutant are regulated by FNR, the main transcriptional regulator of the adaptive response to the lack of oxygen in *S. enterica* ^9,13,16,27,29,30,33,38^. However, this transcriptional regulator was not affected by the presence or absence of *yedF*. We found that in the absence of *yedF*, even when oxygen is available and most of the FNR is inactive^39^, a significant activation of the genes encoding for the anaerobic enzymes occurs, suggesting that *yedF* contributes to the regulation of these enzymes. This was not seen at a protein level, as only the hydrogenase HypB and the fumarate reductase FrdA were detected but did not show differential amounts between the strains studied. These results are expected as protein synthesis of enzymes involved in the anaerobic metabolism is not needed in aerobic conditions.

Dahl *et al*.^16^ proposed a model in which interaction of the IscS protein with TusA decreases the pool of available IscS needed for FeS cluster biogenesis and, consequently, for the activation of FNR. As YedF also contains the same conserved cysteine, the catalytic residue involved in the interaction with IscS^39^, we propose that this may have occurred in the present study. Based on the available information and the results obtained in this work, we propose that *yedF* affects gene activation by influencing FeS cluster biosynthesis in the cell, in the same way as the model previously proposed for TusA^16^. Supporting this statement, in this work we also show that in the wild-type strain SL1344, when oxygen is not available, *yedF* is repressed whereas the anaerobic metabolism-related genes *dmsA* and *frdA* are overexpressed compared to aerobic conditions.

In addition, we propose that *yedF* may have a role in virulence of *Salmonella*. Our results demonstrate a decrease in virulence-related determinants at both gene and protein levels, which correlated with the reduced *in vitro* ability to interact with epithelial cells of the strain lacking *yedF* and was restored when the gene was reintroduced. Virulence regulation is a complex process with many contributing factors leading to gene expression in particular conditions; therefore, the interpretation of the specific role of *yedF* in this system requires further study. Nevertheless, our results indicate that this gene is involved in the regulation of virulence as the absence of *yedF* results in significant changes. Accordingly, we propose this gene to be named *vrf*, for virulence-related factor. Taking into account this multifactorial regulation, an alteration of the signaling network could, in some cases, lead to paradoxical situations, such as the unexpected gene expression profile of determinants involved in the flagellin biosynthesis pathway.

In addition, we found that LPS was also affected by *yedF*. Previously, Kong *et al*.^40^ reported that alteration of the LPS composition in *S*. Typhimurium led to a decrease in virulence in an *in vivo* murine model. In that study, mutants obtained by the deletion of genes involved in the O-antigen and core biosynthesis of LPS, also shown to be transcriptionally repressed in the present work, were administered orally in mice. The study revealed that intact LPS was required for optimal invasion and colonization of host tissues. Accordingly, the effect of *yedF* on LPS composition may partially contribute to the decreased ability to interact with HeLa cells detected in the *yedF*-defective mutant in addition to the proper repression of the invasion genes.

In the present study, we have identified a transcriptional regulator, *yedF*, which activates many genes involved in the host-pathogen interaction. Thus, we propose that *yedF* contributes to the activation of the virulence machinery. Taking into account the pleiotropic functions of *yedF* observed in this work, we hypothesize that *yedF* is activated in the initial steps of infection and contributes to positive transcription of the genes involved in motility and attachment, facilitating the arrival of bacterium to the host cell. In addition, *yedF* may be involved in the activation of genes encoded in SPIs needed for its internalization. When oxygen is available, *yedF* contributes to keeping the anaerobic machinery repressed; while when bacteria encounter conditions in which oxygen is absent, such as in the intracellular environment, transcription of *yedF* is repressed, allowing the activation of the anaerobic metabolism pathway. Nevertheless, further characterization of this factor needs to be undertaken in order to validate this theory.

## Methods

### Clinical isolates and genes selected

In a previous work published by Fàbrega *et al*.^15^, a *S*. Typhimurium clinical isolate and an antibiotic-resistant mutant, 50–64 was obtained. Five unknown genes potentially involved in antibiotic resistance/virulence were selected based on previously performed microarray analyses (>2-fold significant difference)^15^. These genes included three putative inner membrane proteins: *STM1441* (Gene ID: 1252959), *ynaJ* (Gene ID: 1253180) and *ybdJ* (Gene ID: 1252102); one putative outer membrane protein named *ydiY (*Gene ID: 1252845) and one hypothetical transcription factor, *yedF* (Gene ID: 1253487). Sequences were obtained from the S. Typhimurium LT2 reference strain (RefSeq NC_003197.1).

### Construction of Δ*STM1441*, Δ*ynaJ*, Δ*ybdJ*, Δ*ydiY and* Δ*yedF* mutants

Individual inactivation of the genes of interest was done in the reference strain SL1344 following the Datsenko & Wanner method^41^. Strains and primers used are listed in Supplementary Table 1 and Supplementary Table 2, respectively. Selection of the knock-out colonies was made by antibiotic selection using Luria-Bertani (LB) supplemented with 8 mg/L chloramphenicol (Sigma-Aldrich) and 10 mM arabinose (Panreac). Confirmation of the deleted region was done by PCR, and the absence of gene expression of the genes was checked by RT-PCR using specific primers (Supplementary Table 3).

### Obtainment of strains overexpressing *STM1441*, *ynaJ*, *ybdJ, ydiY and yedF*

Constructions of the cloning vector pBAD33 carrying the full genes *STM1441*, *ynaJ*, *ybdJ, ydiY and yedF* were made using their respective primers (Supplementary Table 2). These fragments, as well as the vector pBAD33, were purified and digested with the restriction enzymes XbaI and SacI. The primers used for amplification were pBAD_F (5′-CTGTTTCTCCATACCCGTT-3′) and pBAD_R (5′-CTCATCCGCCAAAACAG-3′) published by Guzman *et al*.^42^. The resulting mutants (Supplementary Table 1) were selected in LB containing 30 mg/L of chloramphenicol (Sigma-Aldrich). Gene expression was induced with 10 mM arabinose (Panreac), and overexpression was confirmed by RT-PCR using the primers shown in Supplementary Table 3.

### Complementation of the expression of *yedF* in the Δ*yedF* mutant

The ∆*yedF* strain with a disrupted *yedF* was complemented in *trans* with p9817, a high copy number plasmid, carrying the gene of interest (∆*yedF*_p9817*yedF*). Both plasmid p9817 and the amplified fragment of the gene of interest containing the restriction enzyme sites were digested with NdeI and BamHI. Confirmation of the recombinant plasmid containing the gene of interest was made by PCR and DNA sequencing using the primers 1681.for (5′-CCCCAGGCTTTACACTTTATGCTTCC-3′) and 1030.rev (5′-GCGGATGCCGGGAGCAGACAAGCCC-3′) at an annealing temperature of 57 °C. Positive colonies were selected in LB containing 50 mg/L of ampicillin (Sigma-Aldrich). The strains obtained in this study are listed in Supplementary Table 1.

### Antimicrobial susceptibility testing

The MICs of nalidixic acid, ciprofloxacin, tetracycline, cefoxitin, erythromycin and trimethoprim were determined by Etests (Biomérieux) on Mueller Hinton-II plates (Becton Dickinson) following the manufacturer’s recommendations. Three replicates of each susceptibility test were performed.

### Gene expression by RT-PCR

RNA of the strains studied was obtained from exponential cultures, in the presence and absence of oxygen, when required, using the Maxwell 16 Research Instrument (Promega) and the Maxwell 16 LEV simplyRNA Blood Kit according to the manufacturer’s recommendations. Then, a two-step RT-PCR was performed in a StepOne Real-Time PCR System (Applied Biosystems) as previously described by our lab^43^. The primers used are shown in Supplementary Table 3. Three independent extractions and analyses were performed.

### Bacterial growth

OD at 620 nm of overnight cultures was determined by means of an iEMS Multiskan Reader MF (Thermo Fisher Scientific) as described previously^15^. Each plate included four replicates of each sample, and the assay was repeated three times.

### Gentamicin protection assay

The *in vitro* assay by bacterial infection of HeLa cells was performed according to Fàbrega *et al*.^15^. When needed, induction of gene expression was done with 10 mM arabinose.

### Endotoxin detection assay

Measurement of the endotoxin content of SL1344_p9817, ∆*yedF*_p9817 and ∆*yedF*_p9817*yedF* was performed in triplicate by means of the kinetic-chromogenic LAL Kinetic-QCL kit (Lonza).

### RNA-Seq

RNA of SL1344 strain and ∆*yedF* was extracted in triplicate as described previously in **Methods**. Then, RNA was quantified using a Quantus Fluorometer (Promega) and integrity was assessed with a 2100 Bioanalyzer (Agilent Technologies). Ribosomal RNA was depleted with the Ribo-Zero Magnetic Kit for Gram-negative bacteria (Epicentre). Then, libraries were generated with the TruSeq Stranded mRNA Sample Prep Kit (Illumina) and sequenced with the Illumina MiSeq platform (2 × 75 bp). An average of 4 million reads/sample was obtained (average Phred quality score = 37). Reads were mapped onto the reference genome (SL1344 NC_016810.1) using EDGEpro software^44^. The resulting count datasets were exported to the DESeq. 2 module in R^45^ for normalization, and pair-wise differential expression was carried out for each gene. Only ± 2 fold change values between both conditions were considered (pvalue < 0.05). Gene Ontology and Pathway analysis according to the KEGG database were conducted with DAVID^46,47^ for all significant genes. Hierarchical clustering of Euclidean distances of the differentially expressed genes was obtained using the pheatmap package in R. Transcriptomic data has been uploaded in the Gene Expression Omnibus (GEO NCBI) Platform (Accession reference “GSE101075”).

### Negative staining electron microscopy

For Transmission Electron Microscopy (TEM) samples of the strains SL1344 and *∆yedF* were prepared from an overnight culture grown aerobically. Pellets were washed twice with PBS, resuspended in 1 mL of a phosphate buffered solution 0.1 M and pH = 7.5 containing 2.5% of glutaraldehyde and kept at 4 °C. Then, a drop of the sample was deposited on a formvar coated copper grid and after 20 min, the excess was removed and a drop of 2% uranyl acetate was deposited for 1 min. Once the grid was dried, observation was done through a TEM JEOL 1010 equipped with a camera CCD Orius (GATAN) at an operating voltage of 80 kV.

### Comparative proteomic analysis iTRAQ

Differences in protein abundance between the SL1344 and *∆yedF* strains were performed using the isobaric tag for relative and absolute quantitation (iTRAQ) technology. Four independent replicas were prepared per strain. Exponential cultures were obtained and cell pellets were finally resuspended in 8 M urea, 2 M thiourea 2.5% 3-[(3-cholamidopropyl) dimethylammonio]-1 propanesulfonate (CHAPS), 2% ASB-14, 40 mM Tris-HCl, pH 8.8. Then, cells were sonicated and the extracted proteins were trypsin digested, and peptides were labeled with 8-plex iTRAQ reagent (ABSciex). Peptides were analyzed by liquid chromatography coupled to mass spectrometry (LC-MS/MS) using a nanoAcquity (Waters) coupled to LTQ-Orbitrap Velos (Thermo Scientific). The Proteome Discoverer software (v1.4) was employed for database search and reporter ion intensities extraction using SequestHT search engine. Further analysis (normalization, protein ration calculation and statistical analysis) were carried out using R Development Core Team/ Inferno RND software. The mass spectrometry proteomics data have been deposited to the ProteomeXchange Consortium via the PRIDE partner repository with the dataset identifier PXD006825.

### Statistical analysis

Non parametrical statistical test consisting in a Kruskal-Wallis rank sum test was performed with R 3.3.3^43,48^. P values ≤ 0.05 were considered as significant.

## Electronic supplementary material

Supplementary information

Supplementary Dataset1
