# Supplementary material for: First insights into the pleiotropic role of vrf (yedF), a newly characterized gene of Salmonella Typhimurium
Source: Sci Rep. 2017 Nov 10;7:15291. doi: 10.1038/s41598-017-15369-7 (PMC5681696; doi:10.1038/s41598-017-15369-7)
Supplement: Supplementary file 1 — Supplementary information [file 41598_2017_15369_MOESM1_ESM.pdf]

Supplementary information:

First insights into the pleiotropic role of *vrf* (*yedF*), a newly characterized gene of *Salmonella* Typhimurium

Clara Ballesté-Delpierre[1], Dietmar Fernandez-Orth[1†], Mario Ferrer-Navarro[1], Ramón Díaz Peña[2], Antonia Odena Caballol[2], Eliandre Oliveira[2], Anna Fàbrega Santamaria[1§*] and Jordi Vila Estapé[1*]

Supplementary Figure 1. Relative differences in the *in vitro* ability of the different strains to interact with HeLa cells. One asterisk indicates a statistically significant difference of p=0.05 in the ability of both STM1441 knock-out strain and STM1441 cloned in pBAD33 compared to the reference strain SL1344.

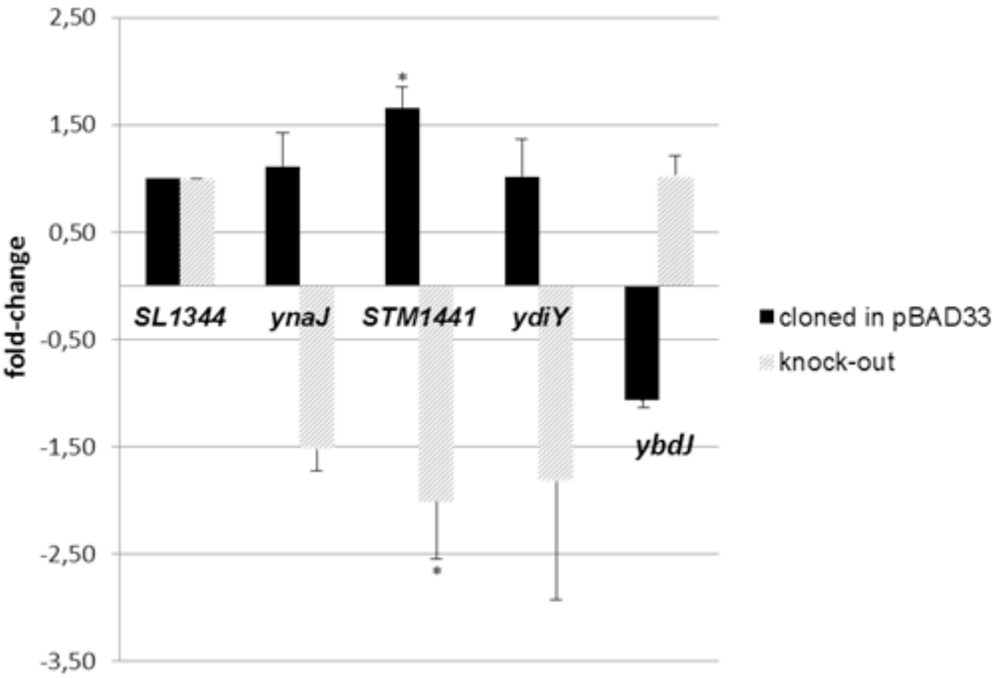

Supplementary Figure 2. Hierarchical clustering of Euclidean distances of the differentially expressed genes between SL1344 and the Δ*yedF* mutant. Expression levels are color-coded from green to red, representing the lowest to highest expression levels in the comparison.

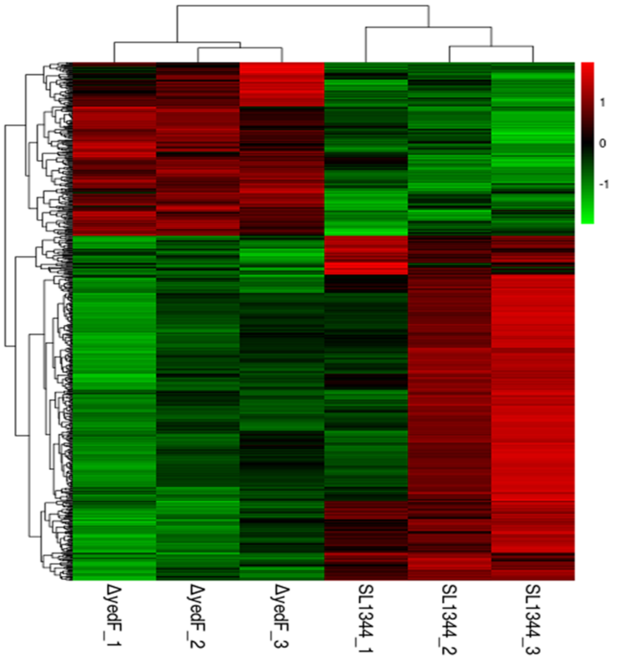

Supplementary Table 1. Strains used in this study. All SL1344-derived mutants were obtained in the present work.

Strains	Description
SL1344	*S. enterica* serovar Typhimurium (wt)
Δ*ybdJ*	SL1344 Δ*ybdJ*::Kan; Kn^{r}
Δ*STM1441*	SL1344 Δ*STM1441*::Kan; Kn^{r}
Δ*ydiY*	SL1344 Δ*ydiY*::Kan; Kn^{r}
Δ*ynaJ*	SL1344 Δ*ynaJ*::Kan; Kn^{r}
Δ*yedF*	SL1344 Δ*yedF*::Cm; Cm^{r}
SL1344_pBAD33	SL1344 harboring pBAD33 empty vector; Cm^{r}
*ybdJ*_pBAD33	SL1344 harboring *ybdJ*_pBAD33 recombinant plasmid; Cm^{r}
*STM1441*_pBAD33	SL1344 harboring *STM1441*_pBAD33; Cm^{r}
*ydiY*_pBAD33	SL1344 harboring *ydiY*_pBAD33; Cm^{r}
*ynaJ*_pBAD33	SL1344 harboring *ynaJ*_pBAD33; Cm^{r}
*yedF*_pBAD33	SL1344 harboring *yedF*_pBAD33; Cm^{r}
SL1344_p9817	SL1344 harboring p9817 empty vector; Ap^{r}
Δ*yedF* _p9817	SL1344 Δ*yedF*::Cm harboring p9817 empty vector, Cm^{r}, Ap^{r}

Δ*yedF* _p9817*yedF*	SL1344 Δ*yedF*::Cm, *yedF*_p9817; Cm^r, Ap^r

Supplementary Table 2. Primers used for cloning and disrupting genes. The annealing temperature used for knock-out obtention was 55ºC in all cases.

Genes	Primers for clonation (5' to 3')	Annealing Temp (°C)	Primers for knock-out (5' to 3')
STM1441	1441.BADF ATATATAGAGCTCAAGGAGAGCGGTAATGAAACTCTC<br>1441.BADR TTTTTTTCTAGAGCGGAATGCGTTAC	56	1441.KOF<br>ACCGTAGGCGGCGTAATCAGTAAAAGTCTGGGGCGTATTGGTGTAGGCTGGA[illegible]<br>GCTTCG<br>1441.KOR<br>AGCGCATCCTGACTATTGCTGAGCTGACTGACGTGATGATCATATGAATATCC<br>CTTAG
ybdJ	ybdJ.BADF ATATATAGAGCTCAAGGAGACAAAGCCATGAAACAC<br>ybdJ.BADR TTTTTTTCTAGACGCATTGAAATGTTTA	53	ybdJ.KOF<br>TTGAATCATTGATGACCGCTGCCGGTATTTTGCTGATGGCGTGTGTAGGCTGG[illegible]<br>CTGCTTCG<br>ybdJ.KOR<br>AATCGGCCAGCGAAAACCAGCGCCGCCAGATAAAGCCATATGAATATCCTCC<br>G
ydiY	ydiY.BADF ATATATAGAGCTCAAGGAGATATATAAATGAAGCTTT<br>ydiY.BADR TTTTTTTCTAGACCGGTACAATATTACATCT	51	ydiY.KOF<br>CAGTACCCGCTGTAGTTATGCTGGCGGGTGGCGTGTTCGCGTGTAGGCTGGA[illegible]<br>GCTTCG<br>ydiY.KOR<br>TTACATCTTATAGCCCAGCGTTACCGTAGTGCGACGATCGCATATGAATATCC<br>TTAG
yedF	yedF.BADF AAATATATGAGCTCCTAAGGAATCTGTATGA (pBAD33)<br>yedF.BADR TTTTTTTCTAGATGCGCTTATTTTTGAA (pBAD33)	53	yedF.KOF<br>ATGAAAAATATCGTCCCTGATTACCGTCTGGATATGGTTGGTGTAGGCTGGA[illegible]<br>GCTTCG
	yedF.CF AAATATATCATATGAAAAATATCGTCCCT (p9817)<br>yedF.CR TTTTTTGGATCCTGCGCTTATTTTTGAATC (p9817)	57	yedF.KOR<br>TTATTTTTGAATCAGATAACGAATTGTCGGGCCATCCTGTTCATATGAATATC[illegible]<br>CTTAG
ynaJ	ynaJ.BADF AAATATATGAGCTCAAAGGACAATACTATGAT<br>ynaJ.BADR TTTTTTTCTAGACCCTATTCCTGTTACT	51	ynaJ.KOF<br>GGTGTGATTGAGCAGTATCATATTCCACTGTCCGAGTGTAGGCTGGAGCTGCT<br>G<br>ynaJ.KOR<br>TTACTCATCTCCGCCGAGGAAATAGATCCCCAAAGCATATGAATATCCTCCTT

Supplementary Table 3. Primers used for RT-PCR.

Genes	Primers for RT-PCR (5' to 3')	Reference
STM1441	STM1441.RT1 TCCCTGCCGGCCCTGCGAAATAC<br>STM1441.RT2 CGATCCAGGCGGCCATGCTAAATA	this study
ybdJ	ybdj.RT1 TGAAACACCCGCTTGAATCA<br>ybdj.RT2 AGCAACGCCATCAGCAAAAT	this study
ydiY	ydiY.RT1 GTATGCCGCCGCCGATGATT<br>ydiY.RT2 TAAGGAACTGCCGCCCGTAAC	this study
yedF	yedF.RT1 CACTGGATGCGCGCAAT<br>yedF.RT2 CGGGCCATCCTGTTGGA	this study
ynaJ	ynaJ.RT1 TATGCCTGCTGGCAGTGTTTA<br>ynaJ.RT2 TCGCGCGCGTTACCA	this study
**Virulence-related genes**		
hilA	hilA_RT_F TTGCTGACTCAATGCGTTAATATG<br>hilA_RT_R TGCCAGCGCACAGTAAGG	[49]
hilD	hilD_RT_F TGGCCACATGGATTTCGATA<br>hilD_RT_R GTGCATAGAGAGCGCCAAGTC	[49]
invA	invA_RT_F TTGGCGATCTCGATAAAGTCTCT<br>invA_RT_R CGGCTCTTCGGCACAAGTAA	[49]
fliC	fliC_RT_F GCCCGTAGCCGTATCGAA<br>fliC_RT_R GCGCGCGAGACATGTTG	[49]
fimA	fimA_RT_F CGCGCAGGTGCCTTTCT<br>fimA_RT_R GGCCGCCACTTTTGGAT	[49]
**Reference gene**		
16S rRNA	16S_RT_F GCGGCAGGCCTAACACAT<br>16S_RT_R GCAAGAGGCCCGAACGTC	[44]

Supplementary Data. List of genes differentially transcribed in SL1344 compared with the Δ*yedF* mutant, considering a p value < 0.05.
